# Convolutional Neural Network-Based Embarrassing Situation Detection under Camera for Social Robot in Smart Homes

**DOI:** 10.3390/s18051530

**Published:** 2018-05-10

**Authors:** Guanci Yang, Jing Yang, Weihua Sheng, Francisco Erivaldo Fernandes Junior, Shaobo Li

**Affiliations:** 1 Key Laboratory of Advanced Manufacturing Technology of Ministry of Education, Guizhou University, Guiyang 550025, China; yang_jing0903@163.com; 2 School of Electrical and Computer Engineering, Oklahoma State University, Stillwater, OK 74074, USA; weihua.sheng@okstate.edu (W.S.); fcoerivaldojr@gmail.com (F.E.F.J.)

**Keywords:** privacy detection, social robot, convolutional neural networks, smart home

## Abstract

Recent research has shown that the ubiquitous use of cameras and voice monitoring equipment in a home environment can raise privacy concerns and affect human mental health. This can be a major obstacle to the deployment of smart home systems for elderly or disabled care. This study uses a social robot to detect embarrassing situations. Firstly, we designed an improved neural network structure based on the You Only Look Once (YOLO) model to obtain feature information. By focusing on reducing area redundancy and computation time, we proposed a bounding-box merging algorithm based on region proposal networks (B-RPN), to merge the areas that have similar features and determine the borders of the bounding box. Thereafter, we designed a feature extraction algorithm based on our improved YOLO and B-RPN, called F-YOLO, for our training datasets, and then proposed a real-time object detection algorithm based on F-YOLO (RODA-FY). We implemented RODA-FY and compared models on our MAT social robot. Secondly, we considered six types of situations in smart homes, and developed training and validation datasets, containing 2580 and 360 images, respectively. Meanwhile, we designed three types of experiments with four types of test datasets composed of 960 sample images. Thirdly, we analyzed how a different number of training iterations affects our prediction estimation, and then we explored the relationship between recognition accuracy and learning rates. Our results show that our proposed privacy detection system can recognize designed situations in the smart home with an acceptable recognition accuracy of 94.48%. Finally, we compared the results among RODA-FY, Inception V3, and YOLO, which indicate that our proposed RODA-FY outperforms the other comparison models in recognition accuracy.

## 1. Introduction

Recent research [1] has shown that the ubiquitous use of cameras and voice monitoring equipment in a home environment raises privacy concerns and affects human mental health; this condition is a major obstacle to the deployment of smart home systems for the care of the elderly and disabled. Furthermore, this condition means that a person who, thinking they are alone, engages in some expressive behavior, such as wild singing, sexual acts, crazy dancing, the discovery of which makes them immediately stop what they are doing [2]. The person feels shame and humiliation, which means that the behavior is something that people are willing to do only if no one else is watching. People need independent space for thinking and behavioral expression. The person would be uncomfortable to be observed for a long time in the home environment [3]. With the rapid development of artificial intelligent technology, many researchers are involved in the study of social robots [4], but these studies are often focused on the development of a better quality of life. However, a social robot is usually equipped with cameras that can witness embarrassing situations faced by their owners, and psychological concerns which have not been fully considered [5]. Moreover, some researchers found that a webcam that can grant remote access to check the situation of home is susceptible to attackers who take advantage of these smart home devices to monitor the user’s [6].

Recent studies showed that the increased use of social robots raises questions on ethics, which have not been considered or were not predictable, and that privacy protection is a critical issue [7]. The study argues that ethical principles should be applied to robotics. Furthermore, some studies pointed out that the design of social robots should consider respect for human autonomy, independence, and privacy [8].

This paper investigates the detection of embarrassing situations for social robots in smart homes using Convolutional Neural Networks. The motivation of this work is to provide a method to lower the risk of a privacy leak. The main contributions of this paper are summarized as follows:

- To protect the sensitive information at the beginning of data collection, we implemented a mechanism for a social robot to detect embarrassing situations and convert privacy information into non-sensitive information.
- We designed an improved neural network structure and feature extraction algorithms based on YOLO and B-RPNs (F-YOLO). We then obtained a robust real-time object detection algorithm based on F-YOLO (RODA-FY) for the social robot.
- We designed six kinds of home situation datasets and verification datasets for training, and three kinds of testing datasets to check the performance of the developed social robot, which included 2580, 360, and 960 pictures, respectively.
- We compared our proposed RODA-FY with Inception V3 network models and YOLO. RODA-FY outperforms other comparison algorithms in terms of predictive estimation.

The rest of this paper is organized as follows. Section 2 reviews the related work. Section 3 introduces the related convolutional neural network (CNN) model and algorithms. Section 4 presents an improved object real-time detection model and feature extraction algorithms. Section 5 describes the hardware platform of the social robot and the object real-time detection algorithm. Section 6 presents the dataset and experimental solution. Section 7 discusses the parameter optimization of the training model. In Section 8, a performance test of the system is conducted, and the results are analyzed. Section 9 displays the compared results. Section 10 concludes the paper and discusses future research issues.

## 2. Related Work

Caine et al. investigated the effect of monitoring devices and behavior of older adults by evaluating the privacy perceptions of participants and their behavior-changing tendencies while being monitored [9]. In their research, older adults interacted with different devices supported by various monitoring technologies, such as cameras, mobile robots, and stationary robots, which have been developed to help elderly people live conveniently in their own homes. The researchers found that the use of monitored devices raised people’s privacy concerns, which caused the users to modify their behavior. Focusing on the development of a fall detection system, Shuai et al. [3] considered a system equipped with physical or psychological disturbance to people’s daily life, that hold sensing devices which are unnoticeable from users; the process of fall detection preserves elders’ privacy. In their research, the recognition process of behavior and activity detection is anticipated to improve the extent of privacy protection of people with respect to cameras. Considering poor privacy in terms of intrusion into the private life of the elderly, Christopher et al. [10] employed an autonomous robot to sustain privacy in assistive environments to improve the acceptance of the surveillance devices. They presented a two-stage strategy and suggested to replace all the human-controlled monitoring devices with a single autonomous mobile system. Its self-assessment provides a possible way to reduce the human factor related to privacy issues, but to ensure the privacy of the elderly, the daily schedule of people and the captured images and videos of camera are not stored on the robot. That information is only sent to the caregiver in case of an emergency. Fischinger et al. [11] developed the Hobbit robot, which is a care robot to support elder adults to independently live at home. Hobbit can prevent and detect falling, and is capable of emergency detection and handling, and provides the function of daily interaction. Hobbit is beneficial to the life of older adults, but in regards to the privacy issue, the designer restricted the actions of the robot, which means that the robot is forbidden to follow the user all the time and to enter bathrooms and toilets.

To better understand the idea of privacy in the smart home environment of elderly people, Shankar et al. proposed a framework based on a questionnaire [12], and refined the proposed framework to obtain the concerns and feedback of the participants to verify designed privacy-sensitive technologies for the elderly. Their research focused on the requirements of the elderly and a definition of privacy that does not address how privacy can be protected. Seo et al. [13] designed a personal privacy protection architecture based on the ISO/IEEE 11073-20601 standard, which can implement communication between health monitoring devices and data managers. The proposed method is designed to provide a more secure and realistic alternative for future human-centric healthcare in smart homes, the framework protects privacy data by delegating various roles with different authorization levels, and its privacy information is limited to the measured health history data, such as pregnancy history and HIV infection. Kozlov et al. introduced an overall architecture for the Internet of Things and analyzed the threats of the attacker, security of system-centric approaches, privacy, and trust from different sub-systems [14]. Classification methods for privacy control mechanisms and privacy levels were proposed, but the methods required stringent law support. Denning et al. held that privacy risk and the associated challenges needed to be addressed while no serious and fundamental security laws for social robotics exist [15]. To explore the potential risk of safety and privacy, this study designed a core set of questions to determine the robot’s influence on the safety and privacy of its owners and their property while the robots were used in the home environment. This study raised some meaningful questions for the ongoing design and evaluation of privacy-respecting robots, and how the use of encryption and authentication technology can protect users’ privacy and security. Sensitive and private information in its original state would be exposed to invaders if an illegal user obtained authentication. Recent research revealed that the use of a distributed control mechanism or an algorithm for decision making and reasoning will compromise privacy through the analysis of the physical layer [16].

The aforementioned studies focus on the protection of private information by employing all kinds of access control technologies. No research has been conducted regarding the conversion of sensitive data to non-sensitive information at the beginning of data collection from various sensors, such as a camera. Nonetheless, deep learning [17] can provide insight into the feature of unlabeled samples and has been applied to speech recognition [18], machine vision [19], motion recognition, [20] and various fields [21,22], which provide a reference for the improvement of privacy detection of social robots.

## 3. Related CNN Model and Algorithms

### 3.1. Deep CNN

Deep CNNs [23] have demonstrated breakthrough performance in some visual tasks, including image classification [24], object detection [25], and other pattern recognition systems [26]. Generally, CNNs are constructed by stacking two types of interweaved layers: convolutional layers and pooling (subsampling) layers. In the convolution operation phase, the weight-sharing structure is used to reduce the number of weights and then to minimize the complexity of the network model. The pooling operation stage involves the use of the image local correlation principle to subsample the feature map and reduce the amount of data processing by extracting feature structure information. In the model training phase, the output feature matrix of the convolution operation is the input of pooling operations, and the result of the pooling operation is the input of the next-layer convolution operation.

#### 3.1.1. Convolution Operation

CNNs employ several local filters to complete the convolutional operation. The local submatrix of input image multiplies the local filter, and its output feature map is used as the convolution output matrix. To improve the performance of the convolution feature extraction, a convolution layer usually has an *n*th local filter of *p* × *p* to output *n* feature map. Generally, the output matrix of the *i*th convolutional operation of the *l*th convolutional layer in the *j*th filter can be calculated as follows:

(1) $$x_{j}^{l,i}=f\left(\underset{j}{\sum }x_{j}^{\left(l-1\right)}*w_{i,j}^{l}+b_{j}^{l}\right)$$

where ***w****^l^_i_*_,*j*_ denotes the weights of the output matrix, and ***b****^l^_j_* represents the bias. * refers to the matrix product. $x_{j}^{\left(l-1\right)}$ denotes the output of the *j*th filter of the (*l* − 1)th convolutional layer. *f*(…) is a nonlinear activation function.

#### 3.1.2. Pooling Operation

The pooling operation is the process of further reducing the size of input data without compromising the inherent correlation of the data. Pooling operations include maximum merging [27], average merging [28], and random merging of means [29]. The input data to the pooling operation is the output of the previous convolutional operation, and the output vector is the input of the convolution operation belonging to the next layer. The output matrix of the *i*th pooling operation of the *l*th pooling layer in the *j*th filter can be calculated as

(2) $$x_{j}^{l,i}=\frac{1}{N}*\left(\overset{n}{\underset{i-1,j-1}{\sum }}x_{j}^{\left(l-1\right),i}\right)$$

where *n* is the number of neurons of the (*l* − 1)th convolutional layer, and $\sum_{i-1,j-1}^{n}x_{j}^{(l-1),i}$ represents the sum of the output matrix of the convolutional operation of the (*l* − 1)th convolutional layer. Our research uses the average merging method to perform the pooling operation.

### 3.2. Object Real-Time Detection Model YOLO

#### 3.2.1. Neural Network Structure of YOLO

**Figure 1:**
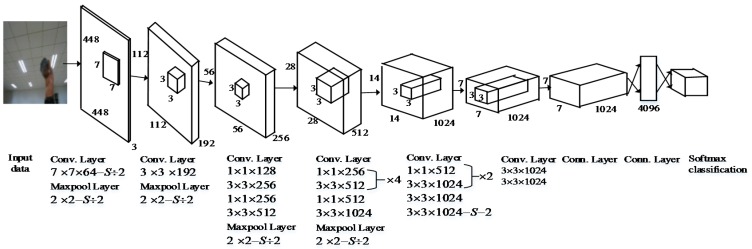
YOLO’s Neural Network Structure.

YOLO [30] is a GoogLeNet model-inspired real-time object detection model proposed by Dr. Joseph Redmon of the University of Washington in 2016. YOLO provides insights globally about the input image and all the objects in the picture. It can then provide end-to-end training and detects objects in real time with reasonably average precision. Figure 1 illustrates the neural network structure of YOLO. The initial convolutional layers of the network response are used to extract features from the input image, and its fully connected layers conclude the output probabilities and coordinates. This network consists of 24 convolutional layers and two fully connected layers. It alternately uses 1 × 1 convolutional layers to reduce the feature space from preceding layers.

YOLO divides the given image into an *S* × *S* grid for object detection. When the center of an object is located in a certain grid cell, the cell responds to detect the object. Each grid cell concludes *B* bounding boxes and the score of each box’s confidence. YOLO employs a five-tuple T (*x*, *y*, *w*, *h*, and *c*) to define the bounding box, where *x* and *y* represent the central coordinates of the box relative to the bounds of the grid cell, *w* and *h* are the width and height predicted relative to the full image, respectively, and *c* is the confidence. Each confidence score indicates how confident the model is that the bounding box includes an object and how precise it determines the box that it foretells. If *P*_0_ is the probability of the box containing one object, and *P*_IOU_ represents the intersection over union (IOU) between the detected object and the forecasted bounding box, then the confidence *c* can be defined as Formula (3). If the cell does not contain an object, *P*_0_ should be zero, and the confidence *c* is zero. Otherwise, if *P*_0_ is one, the confidence *c* can be calculated using Formula (3).

*c* = *P*_0_ × *P*_IOU_ (3)

Assuming that *C* is the number of the class, then the predictions of YOLO are encoded as *S* × *S* × (*B* * 5 + *C*) tensor; *S*, *B,* and *C* were set as 7, 2, and 20, respectively.

#### 3.2.2. Loss Function of YOLO

In YOLO, the loss function $\lambda_{\mathrm{loss}}$ is calculated as

(4) $$\lambda_{\mathrm{loss}}=\overset{S^{2}}{\underset{i=0}{\sum }}E_{\mathrm{coord}}+E_{\mathrm{IOU}}+E_{\mathrm{class}},$$

where *E*_coord_, *E*_IOU_, and *E*_class_ represent the coordinate error, *P*_IOU_ error, and classification error between the predicted data and the calibration data, respectively. The coordinate error *E*_coord_ is calculated as

(5) $$E_{\mathrm{coord}}=\lambda_{\mathrm{coord}}\overset{S^{2}}{\underset{i=0}{\sum }}\overset{B}{\underset{j=0}{\sum }}I_{ij}^{\mathrm{obj}}\left[{\left(x_{i}-{\hat{x}}_{i}\right)}^{2}+{\left(y_{i}-{\hat{y}}_{i}\right)}^{2}\right]+\lambda_{\mathrm{coord}}\overset{S^{2}}{\underset{i=0}{\sum }}\overset{B}{\underset{j=0}{\sum }}I_{ij}^{\mathrm{obj}}\left[{\left(\sqrt{w_{i}}-\sqrt{{\hat{w}}_{i}}\right)}^{2}+{\left(\sqrt{h_{i}}-\sqrt{{\hat{h}}_{i}}\right)}^{2}\right]$$

where $\lambda_{\mathrm{coord}}=5$ is the weight coefficient of *E*_coord_, and *x_i_*, *y_i_*, *w_i_*, and *h_i_* are the predicted information of the grid cell *i*, and ${\hat{x}}_{i},{\hat{y}}_{i},{\hat{w}}_{i},\;\mathrm{and}\;{\hat{h}}_{i}$ are the real information of the grid cell *i*. ${Ι}_{i}^{\mathrm{obj}}\in$ {0,1} denotes whether an object exits in grid cell *i*, and ${Ι}_{i}^{\mathrm{obj}}\in$ {0,1} denotes that the *j*th bounding box predictor in grid cell *i* is “responsible” for that prediction.

*E*_IOU_ is calculated as

(6) $$E_{\mathrm{IOU}}=\overset{S^{2}}{\underset{i=0}{\sum }}\overset{B}{\underset{j=0}{\sum }}I_{ij}^{\mathrm{obj}}{\left(c_{i}-{\hat{c}}_{i}\right)}^{2}+\lambda_{\mathrm{noobj}}\overset{S^{2}}{\underset{i=0}{\sum }}\overset{B}{\underset{j=0}{\sum }}I_{ij}^{\mathrm{noobj}}{\left(c_{i}-{\hat{c}}_{i}\right)}^{2}$$

where $\lambda_{\mathrm{noobj}}$ is the weight of the *P*_IOU_ error, *c_i_* and ${\hat{c}}_{i}$ represent the predicted and real confidence of grid cell *i*, respectively. $I_{i,j}^{\mathrm{obj}}$ ∈ {0,1} denotes that the *j*th bounding box predictor in cell *i* is “responsible” for the non-prediction. $\lambda_{\mathrm{noobj}}$ is set to 0.5 to reduce the transmission error.

The classification error *E*_class_ is calculated as

(7) $$E_{\mathrm{class}}=\overset{S^{2}}{\underset{i=0}{\sum }}I_{ij}^{\mathrm{obj}}\overset{C}{\underset{k=0}{\sum }}{\left(p_{i}\left(k\right)-{\hat{p}}_{i}\left(k\right)\right)}^{2}$$

where and are the conditional probabilities of cell *i* covered by the predicted or real bounding box when cell *i* contains the *k*th class object.

### 3.3. TensorFlow Framewok

TensorFlow is an open-source machine learning framework launched by Google in November 2015 [31]. People can use it to solve various problems with little or no change by integrating TensorFlow with personal systems, such as a PC, large-scale distributed systems, or a high-performance computer with a GPU. TensorFlow also works well on mobile device platforms such as iOS and Android. TensorFlow implements a significant amount of machine learning algorithms, and employs commonly used deep neural network learning models, such as CNN [32], word2vec [33], recurrent neural network [34], and Inception V3 [35]. TensorFlow has been applied in research and deploys machine learning systems into production.

### 3.4. Inception V3 Model Neural Network Architecture

Inception architecture [36], proposed by Google Inc., Mountain view, USA, in 2014, is reputed to be a good deep neural network architecture for computer vision, and was developed to approximate and cover the optimal local sparse structure of a convolutional vision network through available locally dense components. In December 2015, Christian Szegedy et al. proposed the Inception V3 model [35], which is an architecture with improved performance compared to the benchmark, which was applied to object detection, segmentation, human action recognition, video classification, and object tracking. Inceptions V3 is characterized by factorization into smaller convolutions, spatial factorization into asymmetric convolutions, auxiliary classifiers, and efficient grid size reduction.

In Inception V3, the activation dimension of the network filters is expanded to avoid a representational bottleneck before applying maximum or average pooling. Also, the factorization into smaller convolutions is capable of enhancing the space of variations so that the network can provide insight; the use of auxiliary classifiers enables the network to have good accuracy. Inception V3 has a relatively modest computation cost and is a more monolithic architecture. The Inception V3 model has trained networks with a stochastic gradient utilizing the TensorFlow distributed machine learning system.

## 4. Improved Object Real-Time Detection Model and Feature Extraction Algorithm

### 4.1. Origin of Inspiration

We noted that Pedro et al. proposed an object detection system based on the mixtures of multi-scale deformable part models (DPM) [37], which is a typical object detection method, and uses gradient information to extract image features. DPM obtained the gradient model and the object matching relationship by calculating the histogram of the gradient direction to achieve the target classification and detection. DPM divided the potential bounding boxes into grid cell units of the same size, and then extracted the gradient information to weaken the influence of illumination and background. Later, the adjacent cell units were grouped together into the overlapping block to make full use of their information. Then, DPM calculated the entire histogram by normalizing each block’s histogram to reduce the noise effect on the input image. Thereafter, the feature vectors of the whole histogram could be outputted. Finally, the gradient model of classification is obtained by using support vector machines [38]. DPM is capable of reducing the effect caused by background noise and reports good accuracy of classification and recognition.

Region proposal networks (RPN) [17] is a popular object detection method and is a fully convolutional network that simultaneously predicts object bounds and objectless scores at each position, which takes an image (of any size) as the input and then outputs a set of rectangular object proposals. RPN employs the convolutional and pooling operation to extract the feature of the input image, and then uses the bounding box to obtain the feature vector at the last convolutional layer. Finally, it adopts the classification function Softmax to achieve the cost-free classification and region proposals. RPN can reach excellent accuracy of single object recognition in a relatively short time.

The region-based fully convolutional network (R-FCN) [25] consists of the convolutional layer and pooling layer, which employs fully convolutional networks for accurate and efficient object detection. Compared with other CNN-based detection methods, R-FCN conceals a sharing mechanism of image information and shows competitive classification accuracy.

Inspired by DPM, RPN, and R-FCN, when the input data is highly complex and noisy due to illumination, background, and difference of acquisition equipment, and so on, we reference the DPM method to design a new method for YOLO to improve classification and detection performance by increasing the number of grid cell units in the bounding box. Meanwhile, for single or small object detection, we can introduce the RPN into YOLO to achieve better recognition performance. While R-FCN can retain more image information, which is propitious to the extraction of image features, we try to introduce the advantage into YOLO, and then design a CNN-based embarrassing-situation detection algorithm for social robots in smart homes.

### 4.2. Improved YOLO Neural Network Structure

**Figure 2:**
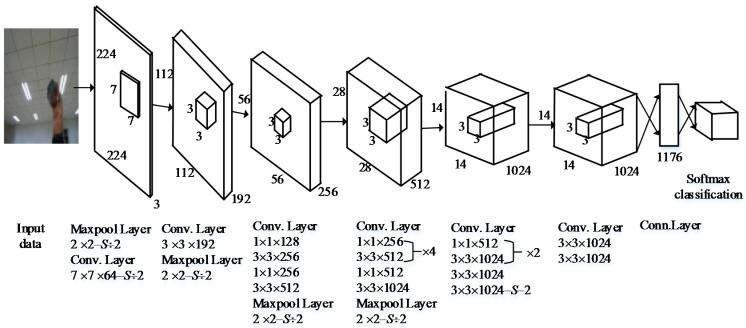
Improved neural network structure based on the You Only Look Once (YOLO) model.

**Figure 3:**
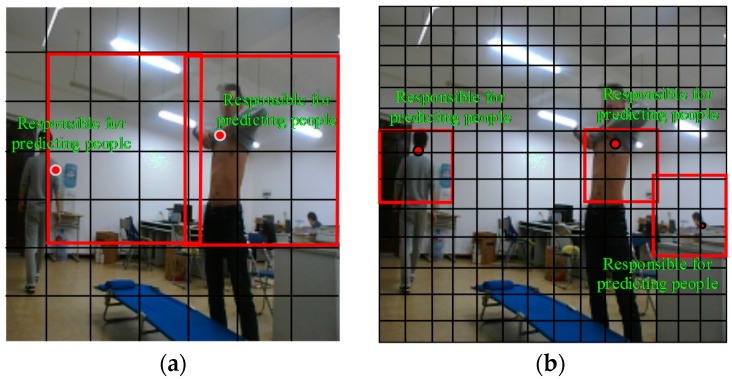
Comparison diagram of object recognition with different grid scale. Subfigure (**a**) and (**b**) are the illustration of 7 × 7 grid and 14 × 14 grid, respectively.

Considering the preceding discussion and inspiration, we designed an improved neural network structure based on YOLO (see Figure 2). This proposed network structure has 24 convolutional layers followed by one fully connected layer. Alternating 1 × 1 convolutional layers reduces the feature space from the preceding layer. The first fully connected layer of YOLO, shown in Figure 2, is deleted by referring to the R-FCN method to reduce the loss of feature information. Referring to the RPN, we increased the size of the 2 × 2 maximum pooling layer to reduce the size of the input image and save the information of the original image. Otherwise, to improve the size of the feature maps, we changed the grid size from 7 × 7 to 14 × 14 after the multilayer convolution and pooling operations. Figure 3 is a comparison diagram of object recognition with different grid scales. As shown in Figure 3, when the grid size is 7 × 7, the system can detect only two objects, while it can report three objects when the grid size is 14 × 14, which is conducive to improving the identification accuracy.

### 4.3. Bounding Box Merging Algorithm Based on RPN

A cell in the YOLO network is associated with multiple bounding boxes, and the final output boxes to identify the object are less than or equal to the image class number *C*. When using the YOLO-based method to recognize privacy situations, not all recognition objects need to be shown, but rather, whether the detected object exists in the current bounding box. Based on such consideration, we designed a bounding box merging algorithm based on RPN (B-RPN), which is detailed in Algorithm 1.

**Algorithm 1:** bounding box merging algorithm based on RPN (B-RPN) **Input:** single image data *X*_pic_ **Output:** bounding box position set *L* of detected object (1) Divide *X*_pic_ into *n* grid cells, and initialize set *R =* {*S*_1_, *S*_2_,…, *S_n_*}, and *L* = Ø; (2) Initialize the similar set *m_i_* of the cell *S_i_* is null, and set the size of bounding box to 14 × 14 specifications; (3)***for***the adjacent area of bounding box pair (*S_i_*, *S_j_*) ***do***  (a) For all the neighbors of *S_i_* in the bounding box, calculate the feature similarity *F*(*S_i_*, *S_j_*) by using RPN;  (b) Find out the maximum similarity *F*_max_(*S_i_*,*S_j_*);  (c) Update the similar set *m_i_* of cell *S_i_*:*m_i_* = *m_i_*∪{ *F*_max_(*S_i_*, *S_j_*)};  **End *for*** (4) ***for*** each *S_i_*
***do***  (a) ***if*** (*m_i_*! = Ø)   (a) Find out all the grid cells corresponding to the elements of *m_i_*, and remove all cells that do not discover an object;   (b) Combine obtained grid cells on the previous step (a) with *S_i_*, and then obtain a new *S_i_*;  (b) *L* = *L*∪{*S_i_*};  **End *for*** (5) Output bounding box position set *L*.  After the convolution and pooling operations, the obtained set *L* was used to merge the areas with similar features and determine the border of the bounding box, which can reduce area redundancy and computation time.

### 4.4. Feature Extraction Algorithm Based on Improved YOLO and B-RPN

This section details the feature extraction algorithm based on the improved YOLO and B-RPN (F-YOLO) for a given training dataset, the pseudocode is shown in Algorithm 2, which combined the improved network structure detailed in Section 4.2 and the proposed B-RPN in Section 4.3.

**Algorithm 2:** Feature extraction algorithm based on improved YOLO and B-RPN (F-YOLO) **Input:** Training dataset *X* of images **Output:** A set of trained weights *M*_weights_ for training dataset *X* (1) Pre-treat images to obtain bounding boxes coordinates. For each image, adopt the soft LabelImg [39] to obtain coordinates of the object that needs to be detected, and then save all images’ coordinate information as file *F*_c_. (2) Load YOLO’s training model of image classification, and initialize *M*_weights_ and coordinates of predicted rectangular area of each image is null; (3) Load file *F*_c_ to generate the matrix-vector set *M*_vec_ of each object’s candidate area of each image through using RPN method; (4) ***for*** each matrix-vector of *M*_vec_ that correspond an image of the training dataset *X **do***  (a) Put the matrix-vector as the input data of the first layer of pooling;  (b) Perform the pooling operation through Formula (2), and put its result as the input data of the next layer;  (c) Employ a bounding box to scan the grid, and use Formulas (1) and (2) to perform convolution and pooling operations to calculate the feature vector of the grid cells, which is located in the bounding box;  (d) The feature vector obtained in the nearest previous step is used as the input of the 18th convolution layer, use Formula (1) to perform the convolution operation based on the R-FCN by using a 2 × 2 stride;  (e) Put the result of step d) as the input of the full connection layer, and carry out the convolution operation by using a 1 × 1 stride;  (f) Apply the classification function Softmax to calculate prediction accuracy probability *P*_pic_ of image *X*_pic_, and output the feature of the object area that corresponding to the largest *P*_IOU_ based on the results of performing the proposed B-RPN by using Formula (3);  (g) According to the probability *P*_pic_, save the obtain feature to the right part of *M*_weights_ ;  **End *for*** (5) Output feature model *M*_weights_;

In step d), the maximum pool layer of 2 × 2, referring to the RPN, used to reduce the size of the image, aims to survive the feature information as much as possible to output a network feature map of 14 × 14. When the convolutional operation is applied, the *P*_IOU_, based on the results of performing the proposed B-RPN, will be substituted into Formula (4) to calculate the minimum value of the loss function. In the later social robot system, feature model *M*_weights_ can be used to recognize different situations in a smart home.

## 5. Privacy Situation Detection Robot Platform and Algorithm

### 5.1. Robot Platform

**Figure 4:**
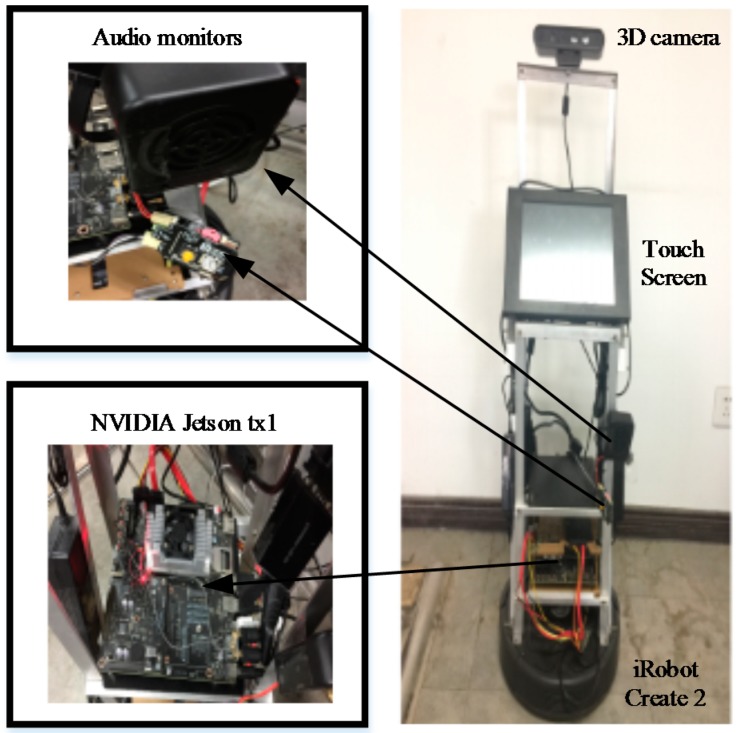
Social Robot Platform of MAT.

Our MAT social robot [40], which used as an experimental platform for conducting research, as shown in Figure 4, was built on an iRobot Create 2 base, Bedford, USA, data acquisition equipment and touchscreen monitor. The display device was a 16-inch industrial touch screen which uses a Linux system. The visual system uses the ORBBEC 3D somatosensory camera (https://orbbec3d.com/) which can capture RGB deep images. The auditory system was based on the expansion of the iFLYTEK voice module which can recognize speech and locate the position of a sound in a noisy environment. The MAT social robot processing system was the NVIDIA Jetson TX1 development board with 256 CUDA cores (http://www.nvidia.cn/object/jetson-tk1-embedded-dev-kit-cn.html). The operating system used was Ubuntu 16.04 with the Robot Operation System (ROS). Data analysis was carried out on a workstation to reduce the computational load of the social robot. At the same time, both the MAT social robot and the workstation were installed with OpenCV 3.1, TensorFlow 0.9, the proposed F-YOLO, and B-RPN.

### 5.2. Real-Time Object Detection Algorithm Based on Improved F-YOLO

**Figure 5:**
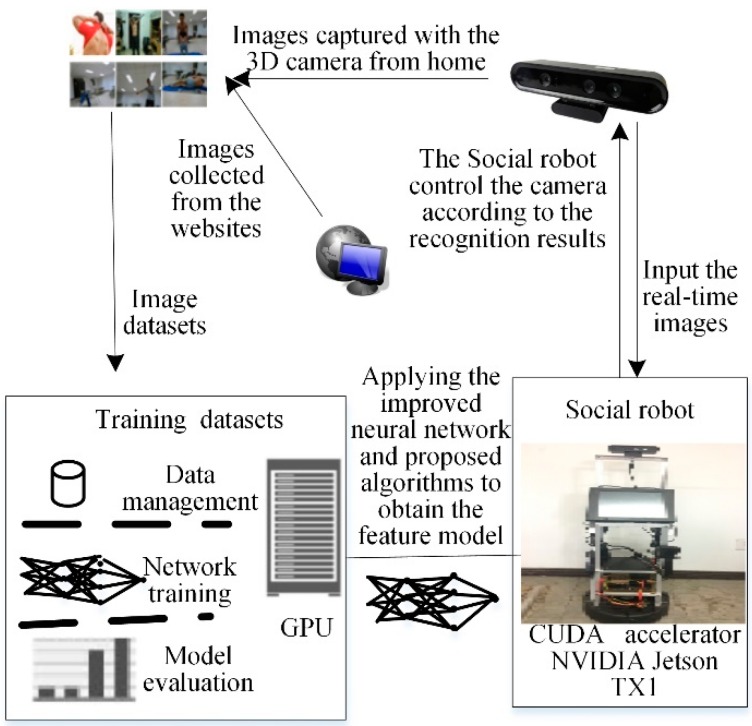
Overall flowchart of privacy situation detection system.

Figure 5 shows the overall flowchart of the privacy situation detection system. The workstation and its GPU used the proposed F-YOLO algorithm to train the training datasets to output the feature model *M*_weights_ of the training datasets. Then, the obtained feature model was downloaded to the MAT robot by the communication pipeline. The MAT robot reads the images from the 3D camera at a given frequency of 10 Hz. The MAT robot needs to make a decision on whether the 3D camera needs to adjust its working model by understanding the images based on the feature model *M*_weights_. Finally, the 3D camera executes the action instruction of the MAT robot. Namely, if a privacy situation is detected the robot turns the camera away from the person and stores the abstract information in a text file according to the detected situations, which is achieved by understanding the context based on the feature model. Thereafter, the MAT social robot tries to recover to the previous state to observe people after receiving a command from the users. If the robot does not receive any command, it will use the speaker to ask the users whether it can observe again. If the reply is negative, the camera keeps turning away from the users; otherwise, the camera focuses on the behaviors of the users until a new privacy situation is detected. Algorithm 3 is the proposed real-time object detection algorithm based on F-YOLO (RODA-FY).

**Algorithm 3:** Real-time object detection algorithm based on F-YOLO (RODA-FY) **Input:** Real-time image *x*_r_ reading from camera;    Feature model *M*_weights_; **Output:** Prediction accuracy probability *P*_r_ of *x*_r_ (1)Load the real-time image *x*_r_;(2)Load feature model *M*_weights_;(3)Generate several matrix vectors of different candidate areas by applying the RPN method on image *x*_r_;(4)Put the matrix vectors as the input data of the first layer of pooling;(5)Perform the pooling operation through Formula (2), and put its result as the input data of the next layer;(6)Employ a bounding box to scan the grid, and use Formulas (1) and (2) to perform convolution and pooling operations to calculate the feature vector of the grid cells, which are located in the bounding box;(7)Use the feature vector obtained in nearest previous step as the input of the 18th convolution layer, and use Formula (1) to perform the convolution operation based on the R-FCN by using a 2 × 2 stride;(8)Put the result of step (7) as the input of the full connection layer, and carry out the convolution operation using a 1 × 1 stride and Formula (1);(9)Apply the classification function Softmax and feature model *M*_weights_ to obtain the prediction accuracy probability *P*_r_ of image *x*_r_;(10)Output the prediction accuracy probability *P*_r_ that is used to predict the class of *x*_r_.


In the algorithm mentioned, Steps (4) to (8) are the processes of feature extraction of the candidate object area and use the proposed B-RPN to obtain the eigenvectors of the maximum merged candidate area. Step (9) employs the Softmax function to finish the feature matching in the obtained eigenvectors and feature model *M*_weights_, and then obtains prediction accuracy probability *P*_r_ of image *x*_r_. According to *P*_r_, the robot system understands the class of detection object, and then the robot executes the proper action based on the above strategy.

For example, on 29 March 8:00 in 2017, the robot detects that the user Simon is taking a shower, the robot then turns the camera away from the person and begins to record the time, and then stores the following information to the file:

29 March 8:00 in 2017, Simon is taking a shower.

After waiting 30 s, the robot uses the speaker to ask the users whether she or he has finished taking a shower. If the reply is negative, the camera keeps turning away from the users. Otherwise, the robot focuses on the behaviors of the users and at the same time records the current time and stores the following information to the file:

29 March 8:15 in 2017, Simon has finished taking a shower.

The robot continues to observe the user’s behavior until the new privacy situation is detected.

## 6. Dataset and Experimental Design

### 6.1. Training Datasets and Validation Datasets

The training datasets consist of an image of different situations in the home, which are used to capture the feature model of the different situations by using the proposed F-YOLO algorithm on the developed MAT robot.

The validation datasets are used to verify the recognition performance of the feature model under various parameters during the process of feature extraction, which can refine the feature model.

We considered six classes of situations in the smart home for the training and validation datasets, shown in Table 1. The training data include the following kinds of images:

(1) The images captured by the ORBBEC 3D camera settled on the MAT robot in the smart home. This kind of image accounts for 81% of the total images.
(2) The images downloaded from different websites account for about 19% of total images. For these kinds of pictures, we considered the diversity of background, objects, light, angles, and pixels.

The training datasets include 2580 samples, in which no repeated data occurs, all the training images are unique, and each class includes 430 images. The validation datasets included 360 different samples in which each class contains 60 images.

### 6.2. Experiment Solution and Test Dataset

To check the performance of the developed privacy situation detection system, we designed three kinds of experiments and four kinds of test datasets, as shown in Table 2. The experiment solutions and test datasets focus on checking the robustness of the developed algorithm when under different persons and backgrounds.

**Figure 6:**
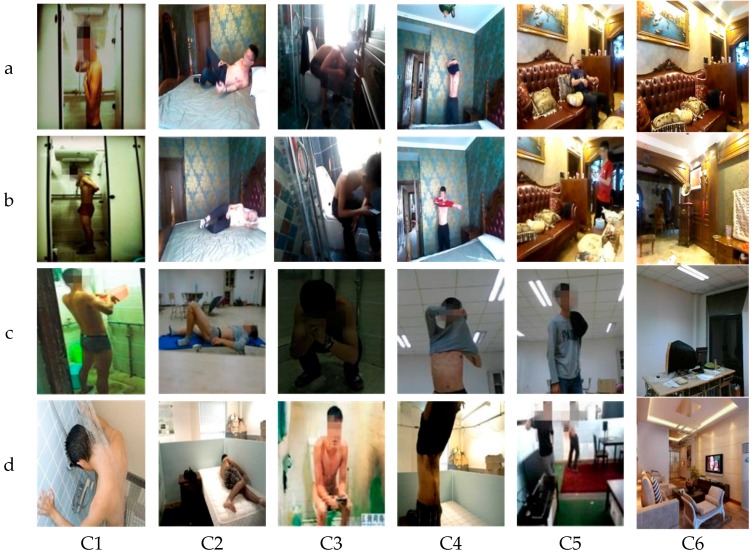
Samples of the used test datasets.

The four kinds of class, a, b, c, and d, included 960 samples, each test category includes 240 sample images, and each situation class includes 40 images. Figure 6 shows a sample illustration of the used test datasets.

The reader can download our datasets from [41].

## 7. Parameter Optimization of Training Model

Considering that executing the proposed F-YOLO to obtain the feature model takes some time and the training epochs have significant effects on the feature model, we studied the influence of different training epochs on the predictive estimation to find out the optimal training epoch. Otherwise, different learning rates have an impact on the recognition accuracy. Thus, we tried to figure out the relationship between the recognition accuracy and the learning rates.

By applying the classification function Softmax, the predictive estimate probability can be calculated by

$$p_{i}^{k}=\frac{\exp\left(\theta_{i}^{k}{}^{T}v_{i}^{k}\right)}{\sum_{k=1}^{K}\exp\left(\theta_{i}^{k}v_{i}^{k}\right)}$$

where *K* is the total classifications number of training datasets, and *n_k_* represents the data size of the *k*th (*k =* 1, 2, ..., *K*) class, and *i* (*i =* 1, 2, ..., *n_k_*) is the label of the *i*th sample in the *k*th class; $v_{i}^{k}$ represents the feature vector of the *i*th sample. $\theta_{i}^{k}$ is the required parameter of the *i*th sample. $\theta_{i}^{k}$ and $v_{i}$ are column vectors.

Besides, the recognition accuracy is the ratio of the number of the correct prediction to the size of the test dataset.

### 7.1. Predictive Estimate Probability Results and Analysis under Different Training Epoch

**Figure 7:**
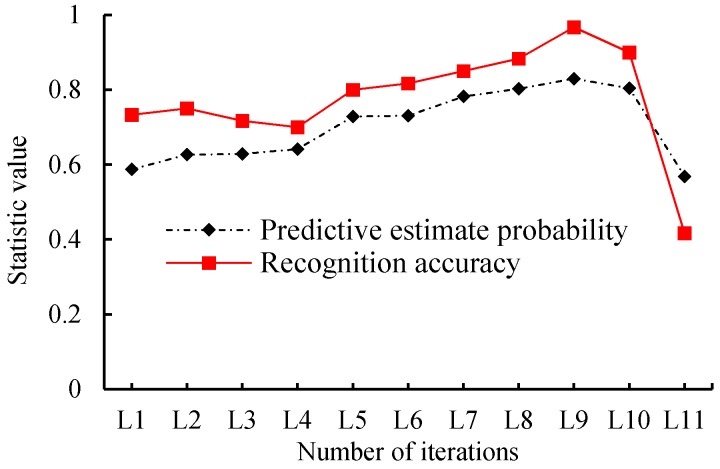
Variation tendency of predictive estimate probability and recognition accuracy with different epochs for validating datasets.

**Figure 8:**
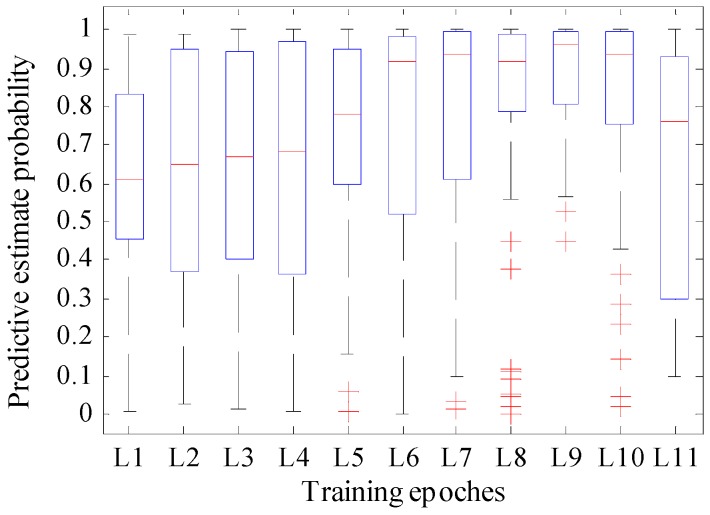
Boxplot of predictive estimate probability with different epochs for validating datasets.

The validation datasets included the 360 unique samples used in this test. We trained the network during a different number of iterations. The detailed epoch and its predictive estimate probability and recognition accuracy of the model are shown in Table 3. Figure 7 shows the variation tendency of the predictive estimate probability and recognition accuracy with different epochs, and Figure 8 is the boxplot of the predictive estimate probability. For this test, the validation data were presented in the previous section used in this test, and the learning rate is set to 0.0001, which is the same as YOLO.

As shown in Figure 7 and Table 3, when the training epoch is 1000, the average prediction estimation probability is 0.588, and the model recognition accuracy is 0.733. With an increase of the training epoch, its prediction estimation probability and recognition accuracy showed an increasing tendency; especially when the training epoch was 9000, the prediction estimation probability reached the highest value of 0.830, and the accuracy was had a maximum of 0.967. Meanwhile, when the training epoch continuously increased to 20,000, the prediction estimation probability dropped to 0.568 with average recognition accuracy of 0.417. We can see that the performance of the model tends to decrease when the epoch is larger than 9000.

According to Figure 8, when the training epoch was between 1000 and 7000, although fewer outliers exist, the rectangle area was longer, and the median line was lower compared with the other epochs. When the training epochs were 8000 and 10,000, although the median line was located at the top half part, many outliers existed, and the prediction estimation probability included a singular point that near zero. When the training epoch was 9000, the rectangular area of the boxplot was narrow with the highest median line compared to the others. Although it still reported outliers, the value of the worst outlier is larger than the value of the normal point of the rectangular regions when the training epochs were 2000, 3000, and 4000. Also, further checking of the corresponding test data showed only two outliers which were greater than 0.45.

Thus, we can conclude that the proposed model showed better performance when the training epoch was set to 9000, and thus, we used this training epoch in the following application.

### 7.2. Relationship between Recognition Accuracy and Learning Rates

**Figure 9:**
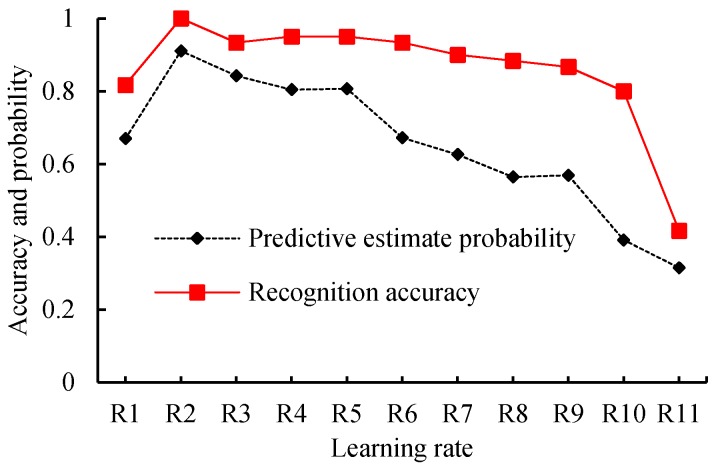
Variation tendency of predictive estimate probability and recognition accuracy with learning rates for validating datasets.

**Figure 10:**
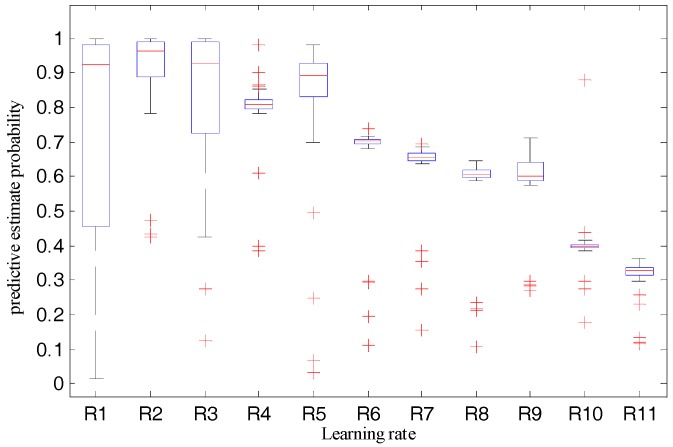
Boxplot of predictive estimate probability with different learning rates.

To find out the relationship between the recognition accuracy and learning rates to obtain a better learning rate that improves the performance of the robot system, we set the training epoch to 9000, and then checked system performance when the learning rates were set to 1, 10^−1^, 10^−2^, 10^−3^, 10^−4^, 10^−5^, 10^−6^, 10^−7^, 10^−8^, 10^−9^, and 10^−10^, respectively. For the validation datasets and training datasets used in this test, we checked the test results of the 360 samples of the validation datasets. Table 4 and Figure 9 and Figure 10 show the statistical results, variation tendency of the predictive estimate probability, and recognition accuracy and the boxplot of predictive estimate probability with different learning rates, respectively.

By observing Figure 9 and Table 4, when the learning rates are greater than 0.1, the average predictive estimate probability and recognition accuracy decrease with the increase of the learning rate. When the learning rates are less than 0.1, the average predictive estimate probability and recognition accuracy decrease with the decline of the learning rate. When the learning rates are arranged at [10^−1^, 10^−4^], the average predictive estimate probability is above 0.8 and the average recognition accuracy is larger than 0.93. When the learning rate decreases from 10^−4^ to 10^−10^, the average predictive estimate probability and recognition accuracy also decrease.

Furthermore, we checked Figure 10. When the learning rate is 1, it has the largest rectangular box area, and its average predictive estimate probability, in Table 4, is only 0.67, but it has a significant probability of being greater than 0.9. This happens because, with bigger learning rates, the algorithm will overshoot around the global minima. When the learning rate is 0.1, although some outliers exist, the rectangular area is narrow, which indicates that the model can steadily output a larger predictive estimate probability. Many outliers exist in the boxplot and there are a large number of outliers with smaller probability when the learning rate is in the range of 10^−10^ to 10^−2^.

Given the preceding evidence, we concluded that the proposed model would show good performance if the learning rate was set to 0.1, and we used this setting for further tests.

## 8. Performance Test Results and Analysis of Proposed System

**Figure 11:**
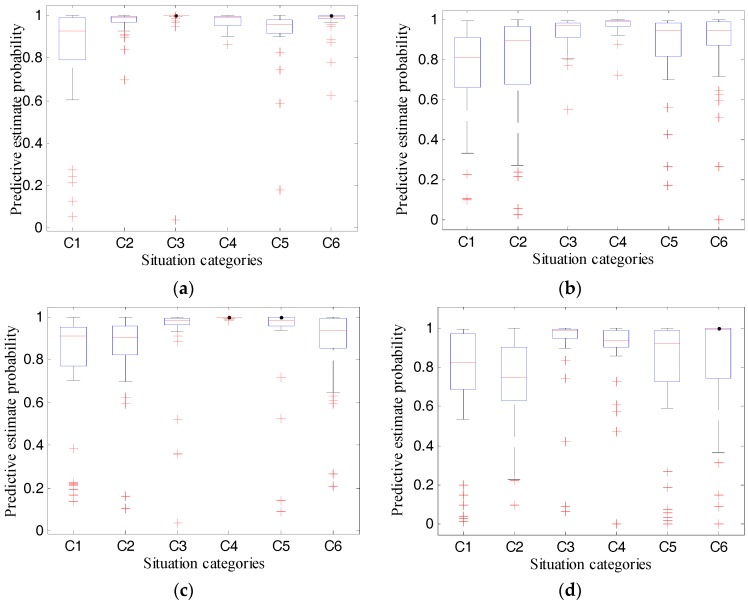
Boxplot of predictive estimate probability. (**a**), (**b**), (**c**) and (**d**) are the results of experiment 1 using test data (**a**) and (**b**), experiment 2, and experiment 3, respectively.

We implemented the proposed RODA-FY with Python and C languages and installed them on the deployed MAT social robot. The learning rate and number of iterations were set to 0.1 and 9000, respectively, and then we finished the four experiments presented in Section 6.2. We observed the test results, and the recognition accuracy of the MAT social robot shown in Table 5, and its predictive estimate probability shown in Table 6 and Figure 11. The results indicate the following.

For experiment 1 using test data a, the average situation recognition accuracies of the system were 0.975 for situations C2, C3, C4, and C6, and the robot was capable of recognizing the situation C5 with a recognition accuracy of 1. However, the system shows poor performance for the recognition of situation C1 with an accuracy of 0.9. When the test data was category b, the MAT social robot performs differently. For situations C2, C3, C4, and C6, the corresponding recognition accuracy rates reported by the robot were 0.950, 0.975, 0.925, and 0.950, respectively. Meanwhile, for situation C5, where persons are in the smart home and do not involve the privacy context C1–C4, the robot can completely recognize this situation. When somebody is taking a shower, the MAT robot reports a situation recognition accuracy of 0.85. According to these two sets of the results, the recognition accuracies decreased by 0.05, 0.025, 0.05, and 0.025 for the situations C1, C2, C4, and C6, respectively. Also, according to Table 6, the system exhibited an average predictive estimate probability of 0.82, 0.968, 0.971, 0.972, 0.920, and 0.972 for situations C1–C6, with standard deviations of 0.275, 0.006, 0.168, 0.038, 0.141, and 0.152, respectively, which indicates that the proposed algorithms are capable of recognizing the given situation with a very large probability when the human, namely the detection object, and the background environments are the same as the training datasets. While for test data b where the human is different from the training datasets, the robot obtained an average predictive estimate probability of 0.789, 0.849, 0.922, 0.977, 0.918, and 0.869 for situations C1–C6, with standard deviations of 0.276, 0.192, 0.096, 0.003, 0.216, and 0.191, respectively, which indicates that the change of the human has an influence on the predictive estimate probability.

For experiment 2 using test data c, the system shows perfect performance with recognition accuracy of 1 in situations C4 and C5, while for situations C1, C2, C3, and C6, the average recognition accuracy rates were 0.850, 0.850, 0.950, and 0.925, respectively. Compared with the results of data a, the rate decreased by 0.05, 0.125, 0.025, −0.025, 0, and 0.05, respectively, which shows that the background environment has an influence on the situation recognition accuracy of the MAT robot. While compared with the results of data b, the rate decreased by 0.0, 0.1, 0.025, −0.075, 0, and 0.025, respectively, which indicates that a change of background environment has more influence on the situation recognition accuracy of the MAT robot than the change of the detection object. Furthermore, compared with the results of test data a and b, the maximum decrease of predictive estimate probability of situations C1, C2, C3, C5, and C6 were 0.069, 0.194, 0.034, 0.066, and 0.108, respectively. This evidence indicates that F-YOLO can obtain a robust feature model with a larger predictive estimate probability to predict the partly-changed new smart home situation by training the limited training datasets, but this change will impede the performance of recognition accuracy of the MAT social robot.

For experiment 3 using test data d, the recognition accuracy of the system was 0.975 and 0.85, but its predictive estimate probabilities were 0.713 and 0.89, these values are smaller than the predictive estimate probabilities of the test data a, b, and c. This condition indicates that both the recognition accuracy and the predictive estimate probabilities decrease when both the background environment and detection objects are not a part of the training datasets. However, test data d consists of various images that are downloaded from websites; their background, object, and camera angles are significantly distinct from the pictures of the training datasets. Meanwhile, the MAT robot can achieve greater than 0.85 recognition accuracy, which shows that the system is robust enough to identify a completely changed new smart home situation.

The system reported 907 correct recognition results of 960 test images; the system exhibited an accurate judgment result of 94.48%, according to all the test results of the testing data. However, by observing Figure 11, we found out that some outliers exist and that the recognition accuracy of some outliers is very small, which means that the system makes the recognition decision under low confidence.

In conclusion, the developed MAT social robot can recognize the designed situations with acceptable recognition accuracy in the smart home. The system shows strong robustness using the obtained feature model to predict a new smart home situation with different backgrounds, objects, or camera angles to the images of the training datasets, which indicates that the proposed RODA-FY can be applied to the social robot to detect privacy situations and provide a foundation for the protection of user privacy.

## 9. Comparison and Analysis

This section provides the comparison results among the proposed RODA-FY, Inception V3 model, and YOLO.

TensorFlow 0.9 implemented the V3 model and provided an interface for users to call this model. The code of YOLO was downloaded from the share link of the authors. We deployed these models into the MAT social robot and called the interface to train the designed training datasets given in Section 6.1. Then, we used the obtained feature model to recognize the new situation. The learning rate and training epochs were set to 0.001 and 9000, respectively.

**Figure 12:**
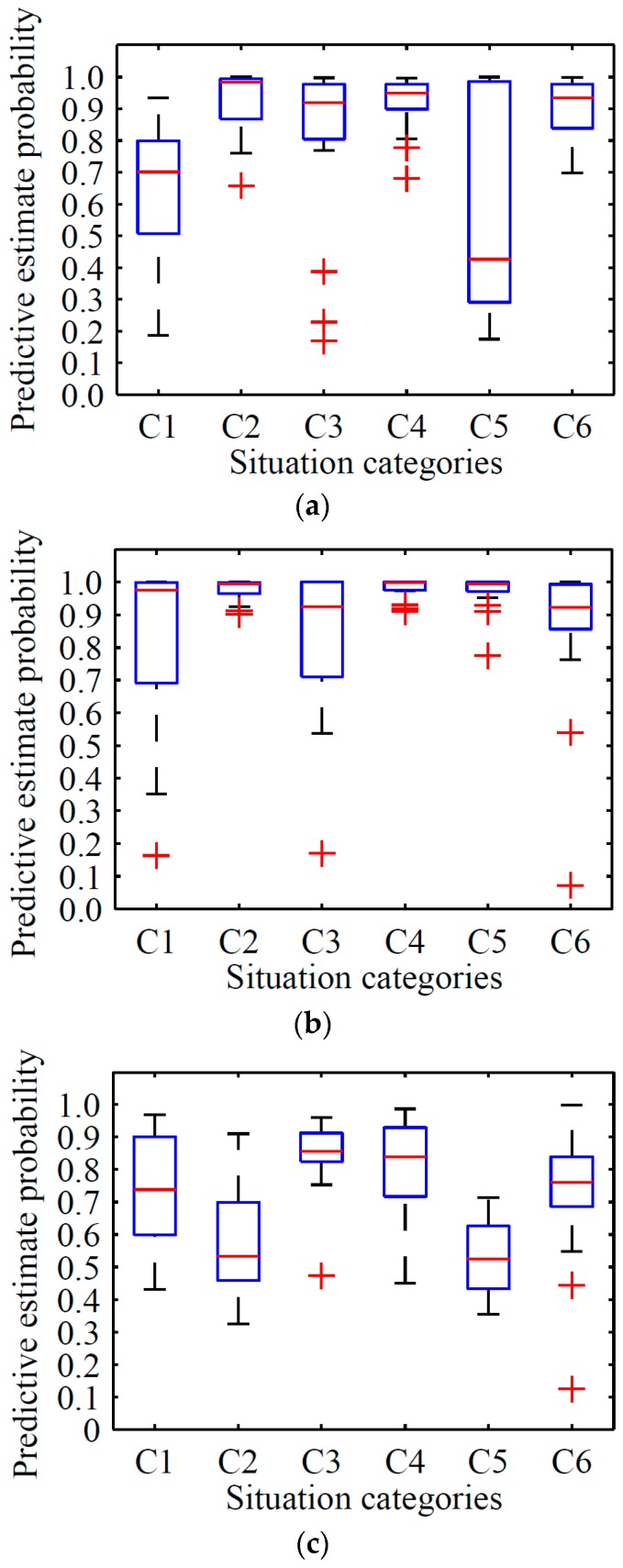
Boxplot of predictive estimate probability obtained by different methods. (**a**), (**b**) and (**c**) are the results reported by Inception V3 model, RODA-FY and YOLO, respectively.

We tested 20 images for each class of test dataset presented in Section 6.2, in which each category contributes 5 images randomly. The test results are shown in Table 7 and Figure 12.

The Inception V3 model showed an average predictive estimate probability of 0.645, 0.929, 0.814, 0.923, 0.576, and 0.972 in situations C1, C2, C3, C4, C5, and C6, respectively, which is less than RODA-FY’s results of 0.131, 0.048, 0.107, 0.048, 0.107, and 0.017, accordingly. With focus on the variance, the results of RODA-FY decreased by 0.008, 0.027, 0.106, 0.154, 0.106, and 0.154 compared with the variance of the Inception V3 model for each respective situation.

The YOLO model shows average predictive estimate probability of 0.741, 0.570, 0.846, 0.814, 0.528, and 0.754 in situations C1, C2, C3, C4, C5, and C6, respectively, which is less than RODA-FY’s results of 0.035, 0.407, 0.075, 0.165, 0.340, and 0.241.

According to the box plot, the proposed algorithm obtained the smallest range with whiskers under the confidence interval. By contrast, the Inception V3 model achieved the largest area with considerable data that were far from the core data. Namely, the results obtained by the Inception V3 model show a more discrete predictive estimate probability, while those from RODA-FY provided more concentrated results. Furthermore, the median line of YOLO stands at a lower position of the range for each situation. Based on the above performances, the proposed algorithm outperforms Inception V3 model in terms of predictive estimation.

We compared the differences of these compared methods. The Inception V3 and YOLO model uses the convolution-then-pooling operation and two full connection layers to insight the feature, whereas RODA-FY only has one full connected layer and the pooling-then-convolution operation applies the proposed F-YOLO algorithm to avoid the loss of feature information. Regarding the feature extraction strategy, the Inception V3 model uses all the area of the training images. However, RODA-FY implemented the mechanism to generate several object candidate areas with different sizes, and then applied the convolution and pooling operations to these areas. We infer that this difference is the intrinsic reason that the RODA-FY outperforms the Inception V3 and YOLO model in terms of predictive estimation.

## 10. Conclusions

In this paper, we concentrated on the privacy issue of the social robot. We designed an improved neural network structure based on YOLO, and proposed the bounding box merging algorithm based on RPN (B-RPN) to achieve improved recognition performance. We described the feature extraction algorithm based on improved YOLO for a given training dataset. We implemented a social robot with the function of privacy situation detection, which employs the proposed real-time object detection algorithm RODA-FY. If a privacy situation is detected, then the robot turns the camera away from the user/s and stores the abstract information in a text file according to the detected situations; this task is achieved by understanding the context of the situation based on the feature model. In our future work, we need to improve the recognition performance of the system, enrich the diversity of the training dataset, and provide better images for the training. Otherwise, it could be a reliable method for obtaining a universal feature model to place those images into training datasets.

## Figures and Tables

**Table 1:** Six kinds of situation in smart home.

Category	Description of Situation
C1	Taking a shower
C2	Sleeping (naked or half-naked)
C3	Using the toilet
C4	Dressing (naked or half-naked)
C5	Humans are in the smart home, and no privacy context is involved
C6	No person in the smart home

**Table 2:** Detailed information on the experiment and test datasets.

Experiment	Purpose of the Experiment	Test Sets		
		Category Name	Numbers of Images	Sample Characteristics
Experiment 1	To check the performance of the developed MAT robot, when the human, namely, the detection object, and the background environments are the same as the training datasets.	a	240	(1) The images captured by the 3D camera; (2) This human and the backgrounds, namely the smart home environment, are included in the training datasets. (3) The images are unique compared with the training datasets;
	To check the robustness of the developed system, when the human is different from the training datasets.	b	240	(1) The images captured by the 3D camera; (2) The backgrounds are included in the training datasets while the human is different from the training datasets.
Experiment 2	To check the performance of the developed MAT robot when the background environments are different from the training datasets, but it is the same human.	c	240	(1) The images captured by the 3D camera; (2) The background environment is distinct from the training datasets while the human is the same as the training datasets.
Experiment 3	To check the performance of the developed system, when the people and the background environment are different from the training datasets.	d	240	(1) Apart of the images captured by the 3D camera, and the others downloaded from the websites; (2) The background environments and person are different from the training datasets;

**Table 3:** Statistical performance with various epochs for validating datasets.

Symbol Name	Epoch	Average Prediction Estimation Probability	Average Recognition Accuracy
L1	1000	0.588	0.733
L2	2000	0.627	0.750
L3	3000	0.629	0.717
L4	4000	0.642	0.700
L5	5000	0.729	0.800
L6	6000	0.731	0.817
L7	7000	0.782	0.850
L8	8000	0.803	0.883
L9	9000	0.830	0.967
L10	10,000	0.804	0.900
L11	20,000	0.569	0.417

**Table 4:** Model performance with different learning rates for validating datasets.

Symbol Name	Learning Rate	Average Prediction Estimation Probability	Average Recognition Accuracy
R1	1	0.670	0.817
R2	10^−1^	0.911	1.00
R3	10^−2^	0.843	0.933
R4	10^−3^	0.805	0.950
R5	10^−4^	0.801	0.950
R6	10^−5^	0.672	0.933
R7	10^−6^	0.626	0.900
R8	10^−7^	0.565	0.880
R9	10^−8^	0.569	0.867
R10	10^−9^	0.391	0.800
R11	10^−10^	0.315	0.417

**Table 5:** Privacy situation recognition accuracy of proposed system for various testing datasets.

Experiment	Category of Test Data	Average Recognition Accuracy with Six Situations					
		C1	C2	C3	C4	C5	C6
Experiment 1	a	0.900	0.975	0.975	0.975	1.000	0.975
	b	0.850	0.950	0.975	0.925	1.000	0.950
Experiment 2	c	0.850	0.850	0.950	1.000	1.000	0.925
Experiment 3	d	0.850	0.850	0.850	0.900	0.975	0.875

**Table 6:** Statistical results of situation predictive estimate probability of the proposed system for different testing datasets.

Category of Test Data	Average Predictive Estimate Probability with Six Situations											
	C1		C2		C3		C4		C5		C6	
	Mean	Variance	Mean	Variance	Mean	Variance	Mean	Variance	Mean	Variance	Mean	Variance
a	0.820	0.275	0.968	0.006	0.971	0.168	0.972	0.038	0.920	0.141	0.972	0.152
b	0.789	0.276	0.849	0.192	0.922	0.096	0.997	0.003	0.918	0.216	0.869	0.191
c	0.751	0.359	0.774	0.253	0.937	0.272	0.974	0.047	0.854	0.212	0.864	0.214
d	0.742	0.304	0.713	0.274	0.854	0.292	0.890	0.186	0.768	0.332	0.807	0.311

**Table 7:** Statistical results of predictive estimate probability using the Inception V3 model and RODA-FY.

Situation	Algorithms	Predictive Estimate Probability	
		Mean	Variance
C1	Inception V3	0.645	0.335
	YOLO	0.741	0.031
	RODA-FY	0.776	0.327
C2	Inception V3	0.929	0.063
	YOLO	0.570	0.028
	RODA-FY	0.977	0.036
C3	Inception V3	0.814	0.305
	YOLO	0.846	0.031
	RODA-FY	0.921	0.199
C4	Inception V3	0.923	0.178
	YOLO	0.814	0.022
	RODA-FY	0.979	0.024
C5	Inception V3	0.576	0.303
	YOLO	0.528	0.012
	RODA-FY	0.868	0.187
C6	Inception V3	0.972	0.077
	YOLO	0.754	0.023
	RODA-FY	0.995	0.013
